# Supplementary figures and images for: ESI mutagenesis: a one-step method for introducing mutations into bacterial artificial chromosomes
Source: Life Sci Alliance. 2020 Dec 7;4(2):e202000836. doi: 10.26508/lsa.202000836 (PMC7756954; doi:10.26508/lsa.202000836)

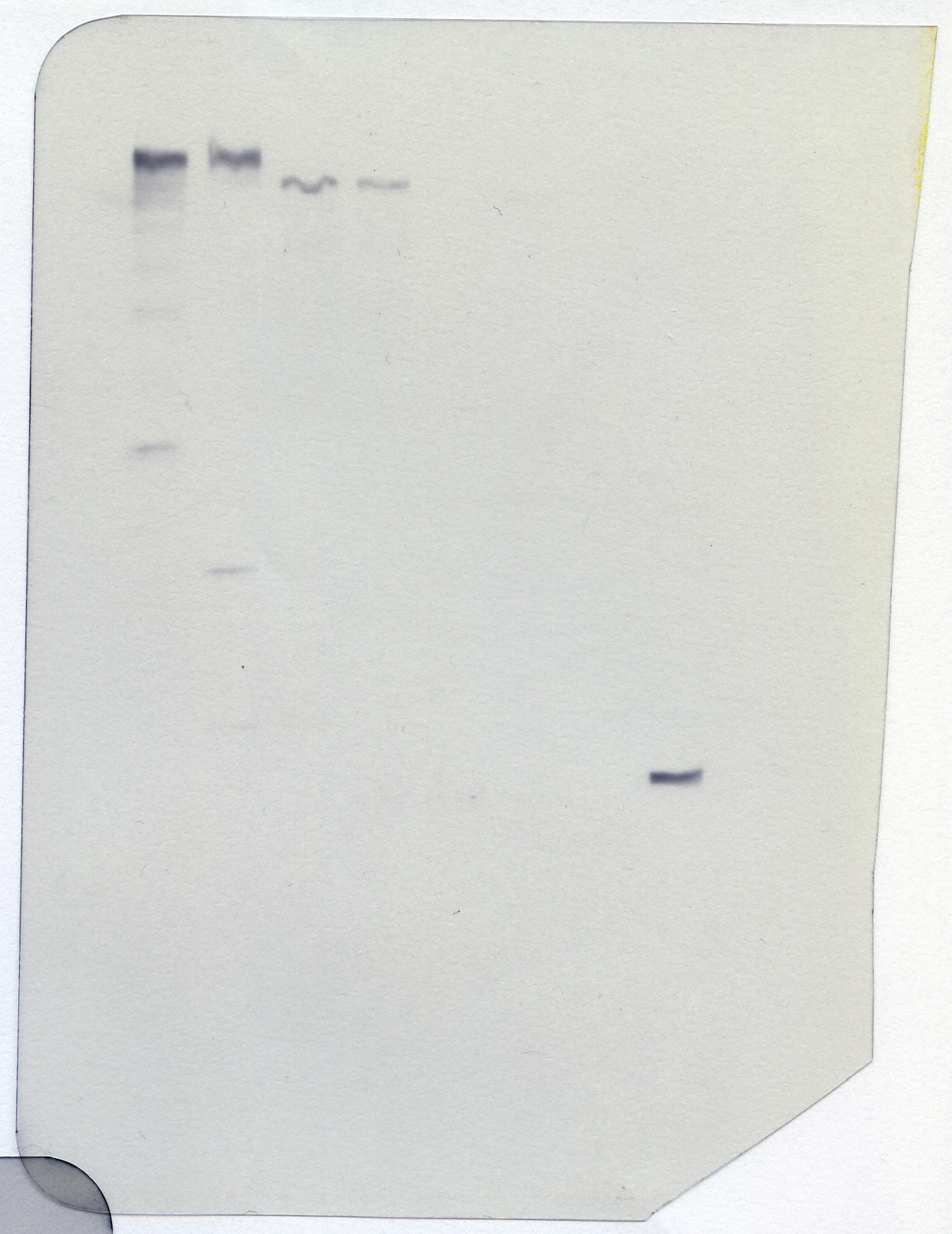

Supplement: Supplementary file 2 [file LSA-2020-00836_SdataF2.1.tif]

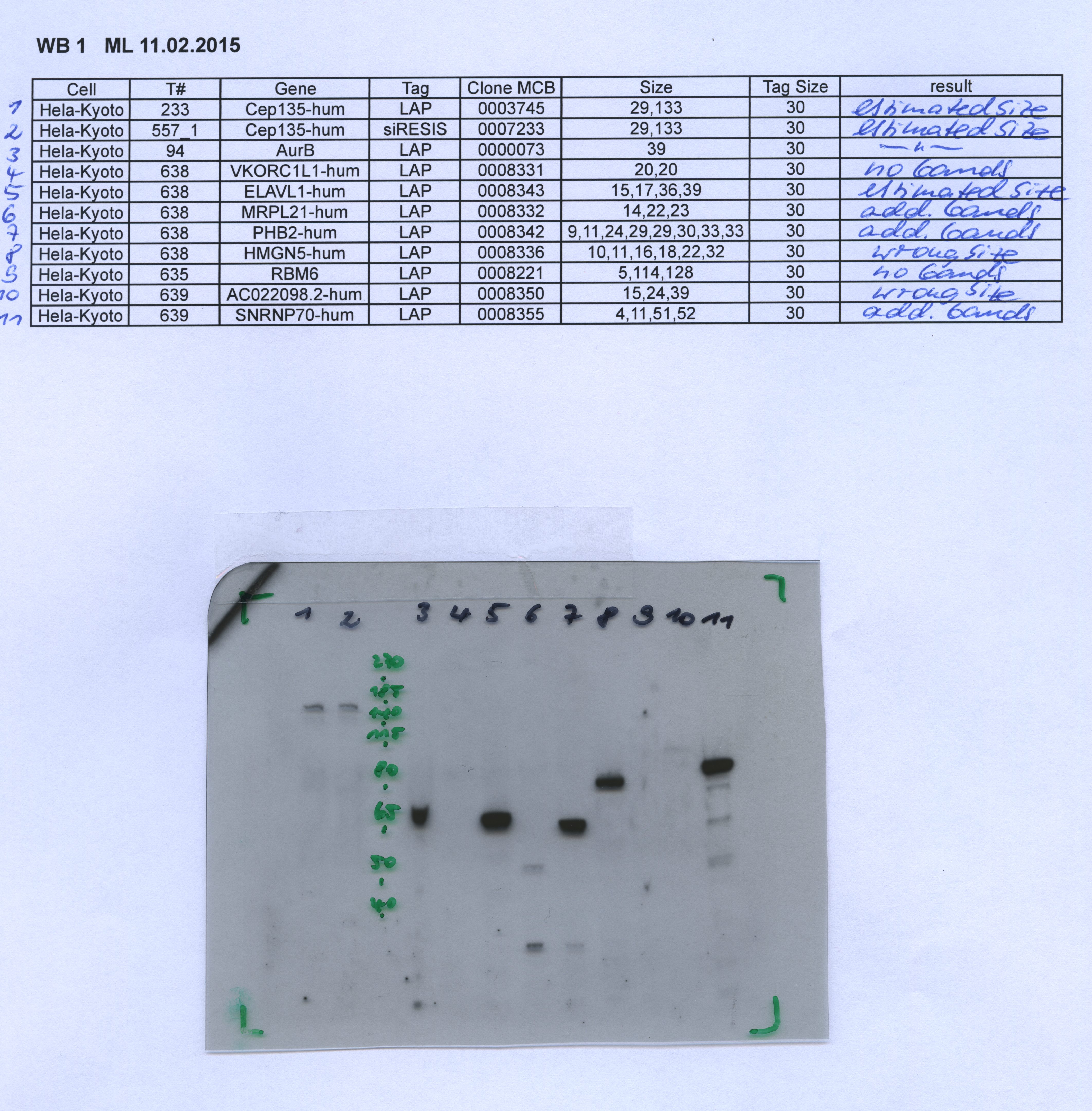

Supplement: Supplementary file 3 [file LSA-2020-00836_SdataF2.2.tif]

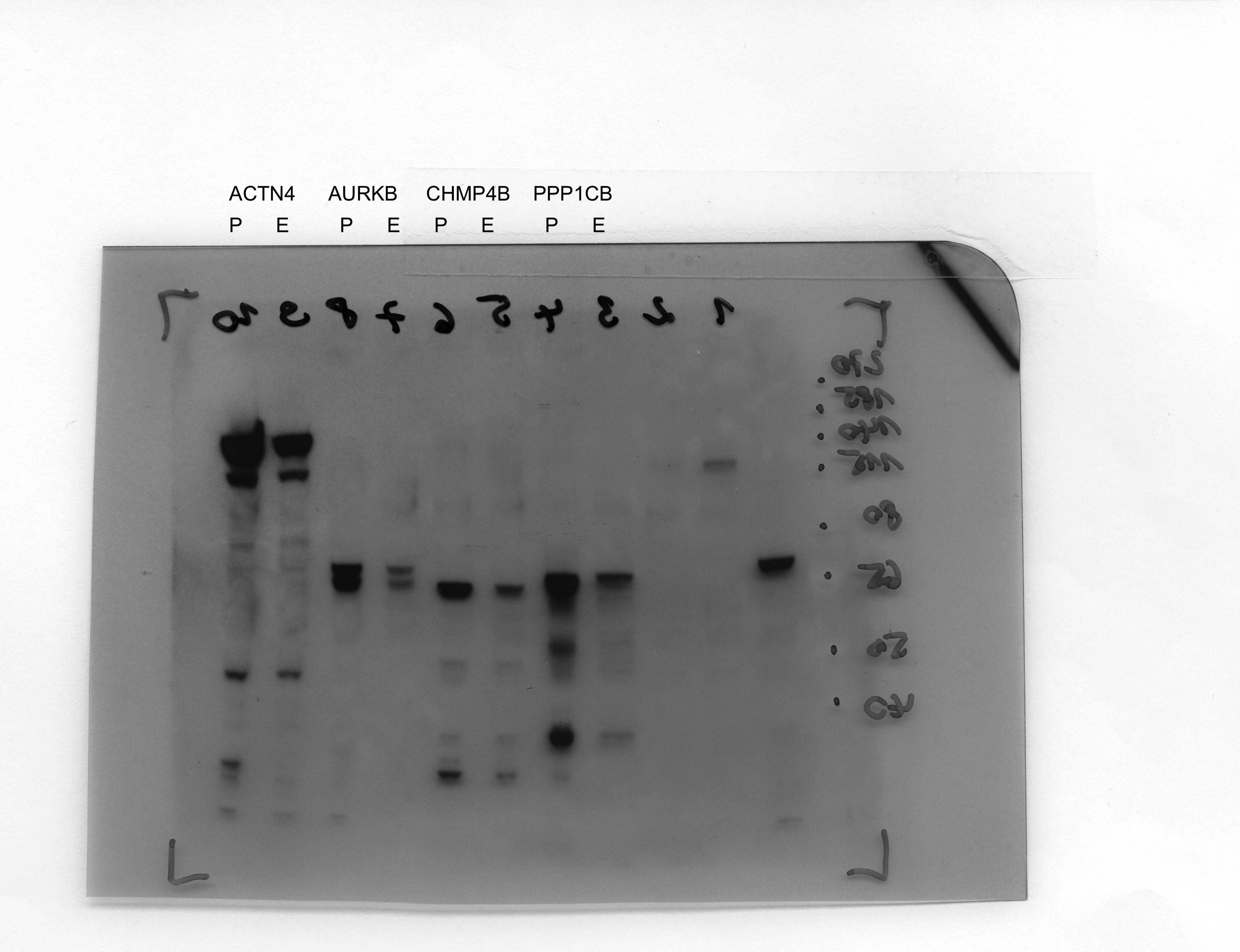

Supplement: Supplementary file 5 [file LSA-2020-00836_SdataF2.4.tif]

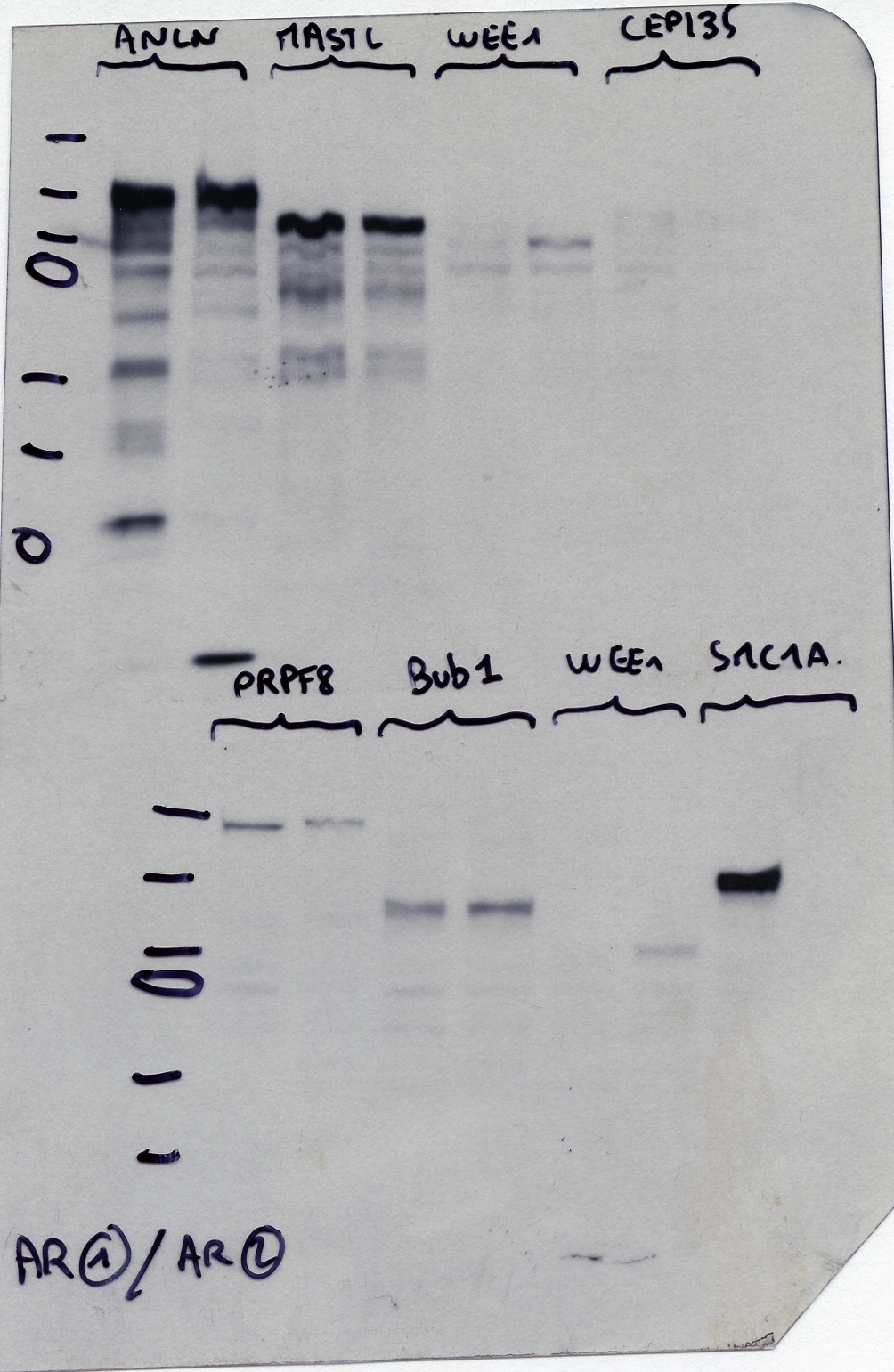

Supplement: Supplementary file 6 [file LSA-2020-00836_SdataF2.5.tif]
