# Supplementary material for: ESI mutagenesis: a one-step method for introducing mutations into bacterial artificial chromosomes
Source: Life Sci Alliance. 2020 Dec 7;4(2):e202000836. doi: 10.26508/lsa.202000836 (PMC7756954; doi:10.26508/lsa.202000836)
Supplement: Supplementary file 7 [file LSA-2020-00836_Supplemental_Data_1.docx]

Supplementary Protocol 1. ESI BAC mutagenesis

BAC selection, ordering, and storage

BACfinder (http://www.mitocheck.org/cgi-bin/BACfinder) will automatically provide a list of BACs containing a given gene of interest (mouse or human), and highlight those on which more than 10kb of genomic DNA are retained on both sides of the gene of interest.

Individual BACs may be obtained from a number of sources, including Children’s Hospital Oakland Research Institute (CHORI) <https://bacpacresources.org/>. They are delivered in a bacterial host and should be stored as bacteria at -80C.

Oligonucleotide design

1. Identify a sequence close to the site to be mutated which can serve as splice site sequences for a new intron. (e.g. (C/A)AGG or similar; see example in Fig S2)

2. Design oligos containing 50bp arms with homology around the targeted site, containing desired mutations close to the new intron site, and if necessary modifying the splice site sequences. 3’ of these sequences attach the sequences for amplifying the AI cassette:

For cassettes used in this study, the corresponding amplification sequences to add are:

Forward oligo: GTAAGTGTGTACGGTGGGATCC (all cassettes)

Reverse oligo: CTGTGGGGAAAAAAAAGTGGATGTTAA (for TH0496, TH0480, and TH0821-based cassettes)

CTGTAAGAGAAAGGAGGTGGATGTTAA (for TH0823-based cassette)

Order these oligonucleotides with HPLC purification

Chemicals

L-arabinose stock solution: 10% L-arabinose in ddH20; freeze aliquots at -20ºC

Antibiotics (as needed)

Chloramphenicol (Cm) stock solution: 30mg/ml in ethanol

Tetracycline (Tet) stock solution: 10mg/ml in 75% ethanol

Kanamycin (Km) stock solution: 40mg/ml in ddH2O

ClonNat stock solutiom 100mg/ml in ddH2O

Zeocin stock solution: 100mg/ml in ddH2O

Liquid Media

LB medium

LB+Cm: LB medium + 15ug/ml Chloramphenicol

LB+Cm+Tet: LB medium + 15ug/ml Chloramphenicol + 3ug/ml Tetracycline

Plates

LB+Cm+Tet: LB medium + 15ug/ml Chloramphenicol + 3ug/ml Tetracycline LB+Cm+Tet+(cassette antibiotic): LB medium + 15ug/ml Chloramphenicol + 3ug/ml Tetracycline + 15ug/ml Kanamycin (or 50ug/ml ClonNat or 25ug/ml Zeocin)

PCR reagents

High-fidelity polymerase

dNTPs

Equipment

PCR machine

Benchtop microcentrifuge

Microcentrifuge tube shaker

Before Starting

Make a DNA prep of pSC101gbaA (tetracycline resistance) (Poser et al, 2008) or similar recombineering plasmid using a standard plasmid purification protocol/kit.

Transform pSC101gbaA into bacteria harboring BAC of interest.

Day 1

Start an overnight culture of bacteria containing the BAC of interest in 1.0 ml LB+Cm in a microfuge tube. Puncture a hole in the lid for air. Incubate at 37°C overnight with shaking.

Day 2

Before starting: Chill ddH2O, electroporation cuvettes and benchtop centrifuge. (Or keep all at 4ºC)

1. Inoculate 1.4ml LB+Cm in a microfuge tube with 30ul of the fresh overnight culture and grow for 2-3 h at 37ºC, shaking at 1000 rpm.

2. Prepare the cells for electroporation: Centrifuge for 30 sec at 11,000 rpm in a cooled microfuge benchtop centrifuge (4ºC). Discard the supernatant by quickly tipping out the supernatant twice, and place the pellet on ice. Resuspend the pellet with 1 ml chilled ddH2O, pipetting up and down three times to mix the suspension. Repeat the centrifugation and resuspend the cells again. Centrifuge and tip out the supernatant once more; 20 to 30 µl will be left in the tube with the pellet. Resuspend cells and keep the tube on ice.

3. Add 1 ul (about 10–100 ng) of pSC101gbaA to the cell slurry in the microfuge tube. Mix briefly and keep on ice. Transfer the cell suspension from the tube to the chilled electroporation cuvette.

4. Electroporate at 1350 V, 10 F, 600 Ohms. This setting applies to an Eppendorf Electroporator 2510 using a 1 mm electroporation cuvette. Other devices can be used, but 1350 V and a 5 ms pulse are recommended.

5. Resuspend the electroporated cells in 1 ml LB medium without antibiotics and return them to the microfuge tube.

6. Incubate at 30ºC for 70 min, shaking at 1000 rpm. (pSC101gbaA will be lost at 37ºC).

7. Using a small loop, plate 30 µl cells on LB+Cm+Tet plates. Incubate the plates at 30ºC overnight.

Integrate mutation and AI cassette into site of interest

Day 3

1.Generate the AI cassette with homology ends and mutations by PCR by setting up three tubes with the following reaction:

0.4µl template DNA (0.1-1.0 mg/ml)

2.5µl “Cassette Homology Arm/Mutation Forward” primer (10uM)

2.5µl “Cassette Homology Arm/Mutation Reverse” primer (10uM)

1.0µl dNTPs (10mM)

5.0µl 10x polymerase PCR Buffer

0.5µl polymerase (high fidelity)

38.1µl H2O

------

50.0µl (total volume)

Use the following program to start:

94º 3min

followed by 8 cycles of:

94º 1min

58º 1min

72º 2min 30sec

followed by 15 cycles of:

94º 1min

64º 1min

72º 2min 30sec

and a final elongation:

72º 7min

4º (hold)

(conditions should be adjusted as necessary due to differences in primer sequences, etc.)

2. After checking for product on an agarose gel, combine the three reactions/products, purify using a standard PCR-purification kit, and concentrate by resuspending all in 50µl ddH20 final.

3. Set up overnight cultures by picking colonies from the pSC101gbaA transformation plate and inoculate one microfuge tube containing 1.0 ml LB+Cm+Tet. Puncture a hole in the lid of the tubes for air. Incubate the cultures while shaking at 30ºC overnight.

Day 4

Before starting: Chill ddH2O, electroporation cuvettes and benchtop centrifuge. (Or keep all at 4ºC)

1. Set up two microfuge tubes containing fresh 1.4 ml LB+Cm+Tet and inoculate with

30µl of fresh overnight culture. (one will serve as a “no DNA” control).

2. Culture for about 2 h at 30ºC, shaking at 1100 rpm until OD600 = ~ 0.3.

3. Induce expression of pSC101gbaA genes: Add 50µl of 10% L-arabinose (final concentration 0.3–0.4%). Incubate at 37ºC with shaking for 50 minutes.

4. Prepare the cells for electroporation: Centrifuge for 30 sec at 11,000 rpm in a cooled microfuge benchtop centrifuge (4ºC). Discard the supernatant by quickly tipping out the supernatant twice, and place the pellet on ice. Resuspend the pellet with 1 ml chilled ddH2O, pipetting up and down three times to mix the suspension. Repeat the centrifugation and resuspend the cells again. Centrifuge and tip out the supernatant once more; around 30 µl will be left in the tube with the pellet. Resuspend cells and keep the tube on ice.

5. Add 2-3 µl of the AI cassette purified PCR product to the cell slurry to one of the microfuge tubes and pipette the mixture into the chilled electroporation cuvettes.

6. Electroporate at 1350 V, 10 F, 600 Ohms. This setting applies to an Eppendorf Electroporator 2510 using an electroporation cuvette with a slit of 1 mm. Other devices can be used, but 1350 V and a 5 ms pulse are recommended.

7. Add 1 ml LB medium without antibiotics to the cuvette. Mix the cells carefully by pipetting up and down and pipette back into the microfuge tube. Incubate the cultures at 37ºC with shaking for 70 minutes. Recombination will now occur.

8. Streak the cultures with a loop onto LB+Cm+Tet+(relevant antibiotic for the cassette) plates. Incubate the plates at 37ºC overnight. The pSC101gbaA plasmid will now be lost prior to BAC DNA preparation.

Day 5

1. Pick colonies to simultaneously restreak, and perform colony PCR on candidate mutants. There should not be any colonies on the ‘no DNA’ plate. Add a small amount of each bacteria to the following reaction:

1.5µl “checking-up” primer (10uM)

1.5µl “checking-down” primer (10uM)

0.6µl dNTPs (10mM)

3.0µl 10x polymerase PCR Buffer

0.3µl polymerase (high fidelity)

23.1µl H2O

------

30.0µl (total volume)

Use the following program to start:

94º 3min

followed by 25 cycles of:

94º 1min

59º 1min

72º 3min

and a final elongation:

72º 7min

4º (hold)

(conditions should be adjusted as necessary due to differences in primer sequences, etc.)

Correct integrants should have a PCR product that is approximately 1.3-1.9 kb larger (depending on the cassette) than the product resulting from an unmodified BAC (without other products).

2. PCR products from correct integrations can now be purified and sequenced to verify the presence of the desired mutation without other mutations.
